# The effect of education on adolescents’ knowledge of menstrual characteristics and treatment-seeking behavior

**DOI:** 10.1590/1806-9282.2025003

**Published:** 2025-06-16

**Authors:** Ferdane Koçoğlu, Hilal Kurt Sezer, Semra Kocaöz

**Affiliations:** 1 Niğde Ömer Halisdemir University, Zübeyde Hanım Faculty of Health Sciences, Department of Nursing – Niğde, Turkey.; 2 Niğde Ömer Halisdemir University, Zübeyde Hanım Faculty of Health Sciences, Department of Public Health Nursing – Niğde, Turkey.; 3 Niğde Ömer Halisdemir University, Zübeyde Hanim Faculty of Health Sciences, Department of Pediatric Health and Diseases Nursing – Niğde, Turkey.; 4 Niğde Ömer Halisdemir University, Zübeyde Hanim Faculty of Health Sciences, Department of Obstetrics and Gynecology Nursing – Niğde, Turkey.

**Keywords:** Adolescent, Knowledge, Menstruation, Help-seeking behavior

## Abstract

**OBJECTIVE:**

The aim of this study was to determine the effect of education on adolescents’ knowledge of menstrual characteristics and treatment-seeking behaviors.

**METHODS:**

This quasi-experimental study was conducted on a total of 509 adolescents. Before and after the education, data were collected with structured questionnaire forms at 3-month intervals. The average age of the adolescents who participated in our study was 15.75±1.28 years.

**RESULTS:**

It was found that the treatment-seeking behavior of adolescents who stated that they had heavy and very heavy menstrual bleeding increased significantly after the education. Except for the duration and perception of menstrual bleeding and the number of days of heavy menstruation, those who reported that their menstrual bleeding deviated from normal in all other cases were significantly more likely to consult a physician.

**CONCLUSION:**

It was determined that the education given to adolescents increased their awareness of the menstrual characteristics and treatment-seeking behaviors of those who reported heavy and very heavy menstrual bleeding.

## INTRODUCTION

In the 2 years after menarche, irregularities in the duration, frequency, and quantity of menstruation due to hypothalamic–pituitary–ovarian immaturity lead to abnormal uterine bleeding^1^. Among the causes of abnormal uterine bleeding in adolescents, amenorrhea and HMB are common menstrual disorders^1,2^. Menstrual disorders have a negative impact on adolescents’ health, academic success, and extracurricular activities^3,4^. The phenomenon of avoiding health-seeking behavior, especially due to abnormal menstrual problems, is a common situation among adolescents on a global scale^5^. The incidence of menstrual disorders in adolescents varies between 23 and 76%^6,7^.

It has been indicated that factors that negatively affect adolescents’ treatment-seeking behavior include embarrassment, fear, lack of knowledge, or being unprepared for menstruation^8^. Factors such as social prohibitions on menstrual cycles, parental attitudes, region of residence, and education level may reduce adolescents’ access to accurate information^9^. Almost half of the adolescent girls experience menstruation without any knowledge about it and most of them are afraid to seek help for fear of stigmatization^10^.

It has been reported that adolescents’ reluctance to seek treatment may cause delays in the diagnosis and treatment of menstrual disorders^7^. Studies have focused on menstrual hygiene and practices and health behaviors in adolescents^11,12^, but no study has been found to examine the effect of education on menstrual characteristics and treatment-seeking behaviors in cases of deviation from normal. This study was conducted to determine the effect of education on adolescents’ knowledge of normal menstrual characteristics and their treatment-seeking behaviors in cases of deviation from normal.

## METHODS

### Participants

The research is a quasi-experimental design study with pre-test post-test design (pre-test: December 29–30, 2022/post-test: April 10/14, 2023). The population of the study comprised female students between the ages of 14 and 17 years studying in 17 high schools located in a provincial center in the Central Anatolia region of Turkey. Among these high schools, one high school was selected according to the convenience sampling method. During the period when the study was conducted, a total of 548 adolescents were studying in this high school. Inclusion criteria were (i) had menarche, (ii) consent to participate in the study, and (iii) parental consent for the study. Exclusion criteria were (i) never menarche, (ii) refusing to participate in the study, (iii) not attending the education, and (iv) incomplete completion of the data collection tool. The study was completed with 471 adolescents (86% of the sample) who met the inclusion and exclusion criteria, received the education, and completed the forms fully.

### Data collection tools

Two different structured questionnaire forms prepared by the researchers in line with the literature^4,13,14^ were used before and after the education. The questionnaire form used before the education included a total of 35 questions inquiring sociodemographic characteristics and characteristics related to the menstrual period and therefore the status of consulting a physician. After the education, they were asked about the intensity and duration of menstrual bleeding, its frequency, and the complaints they experienced, and then they were asked to indicate whether the conditions related to menstrual age, duration, amount, and frequency were normal or not in the questionnaire form. They were also asked whether they had consulted a physician for heavy/very HMB or any menstrual complaints after the education.

### Data collection

One week after the pre-test data were collected, a 40-min face-to-face educational session via PowerPoint presentation (anatomy of the female reproductive organ, characteristics of menstruation, related myths, possible problems and solutions or suggestions, deviations from normal, institutions, and contact numbers^15,16^) was conducted outside of class hours. After the education, the adolescents’ questions were answered, a brochure was distributed, and post-test data were collected for 3 months. Data were collected by protecting the privacy of the students and creating an environment that would prevent them from being affected by each other.

### Statistical analysis

In the study, data were entered into SPSS 24.0 IBM program and evaluated. In the post-test after the education, students were asked to rate menstrual characteristics as normal or abnormal. According to the two groups formed, the status of consulting a physician was coded as the dependent variable and menstrual characteristics as the independent variable. The chi-square test was used to compare categorical data. The McNemar test was used to compare the treatment-seeking behaviors of adolescents before and after the education. In all analyses, p<0.05 was accepted as the significance level.

### Ethical issues

In this study, the principles of the Declaration of Helsinki were followed and voluntary participation was ensured. Before starting the study, the necessary permissions were obtained from Niğde Ömer Halisdemir University University's Ethics Committee (No. 2022/12-X), Niğde Provincial Directorate of National Education, and the high school administration where the study was conducted.

## RESULTS

The mean age of the adolescents who participated in our study was 15.75±1.28 years. It was determined that 61.5% of the adolescents received information before menstruation and that they received this information mostly from their mothers (88.7%).

Notably, 98.5% of the adolescents reported menarche between the ages of 11 and 16 years, 82.9% reported menstrual cycles between 21 and 45 days, 55.5% reported regular menstrual cycles, and 87.3% reported menstruation between 3 and 7 days. Nearly 50.2% of the adolescents reported severe and very severe dysmenorrhea, 28.9% reported heavy and very HMB, 83.9% reported HMB between 3 and 10 days, and 26.8% reported changing pads usually and always at intervals of less than 2 h.

Except for knowing two situations that deviate from normal, the rate of adolescents knowing that all other characteristics are normal was over 70%. The percentage of those who did not know that they had not had menstrual bleeding for more than 3 months after menstrual bleeding became regular and that their breast milk discharge outside of pregnancy and puerperium is deviated from normal was 56.5 and 90.9%, respectively (Table 1). Before the education, 23 adolescents applied to a physician due to heavy and very HMB, and after the education, this number increased significantly to 83 (p<0.001). The number of adolescents applying to a physician due to complaints experienced before and after menstruation was 121 before the education, and this number decreased significantly to 63 after the education (p<0.001; Table 2). The difference between the duration of menstrual bleeding, the perception of menstrual bleeding as heavy or very heavy, the number of days of this intensity (p>0.05), and the status of consulting a physician due to menstrual complaints in individuals with abnormal menstrual characteristics after the education compared to before the education was found to be statistically significant (p<0.05; Table 3).

**Table 1:** Adolescents’ knowledge of the normality of menstrual characteristics after education.

Variables (n=471)	Know		Do not know	
	N	%	n	%
Menstrual age	424	90.0	47	10.0
Menstrual cycle	390	82.8	81	17.2
Menstrual pattern	353	74.9	118	25.1
Menstrual bleeding duration	404	85.8	67	14.2
Perceived amount of bleeding	386	82.0	85	18.0
Perceived menstrual pain of bleeding	345	73.2	126	26.8
Changing pads (less than 2 h)	354	75.2	117	24.8
Clot passage in the menstrual cycle	334	70.9	137	29.1
Using double pads	376	79.8	95	20.2
Blood overflow from pads into the bed/clothes	341	72.4	130	27.6
Having complaints on days 4–13 of menstruation	339	72.0	132	28.0
Not menstruating for more than 3 months after menstruation becomes regular	205	43.5	266	56.5
Breast milk flow	43	9.1	428	90.9

**Table 2:** Status of adolescents’ visits to a physician before and after education.

Pre-education	Post-education			Test statistics*
Visiting a physician due to heavy/very heavy menstrual bleeding	Yes	No	Total, n (%)	
Yes	3	20	23 (4.9)	χ^2^=34.81 **p**≤**0.001** **
No	80	368	448 (95.1)	
Total, n (%)	**83 (17.6)**	388 (82.4)	471 (100)	
**Visiting a physician due to symptoms before and during menstruation**				
Yes	16	105	121 (25.7)	χ^2^=21.38 **p**≤**0.001** **
No	47	302	349 (74.3)	
Total, n (%)	**63 (13.4)**	407 (86.6)	470 (100)	

* McNemar test was used.

** p<0.05, %: percentage.

Statistically significant values are denoted in bold.

**Table 3:** Status of adolescents’ pre- and post-education physician visits according to their menstruation-related abnormalities.

Variables		Pre-education				Post-education				p
		Visiting a physician		Not visiting a physician		Visiting a physician		Not visiting a physician		
		n	%	n	%	n	%	N	%	
Menstrual cycle										
	Abnormal (less than 21 days to more than 45 days)	6	7.4	75	92.6	18	22.2	63	77.8	χ^2^ **=0; p=0.023** *
Menstrual pattern										
	Abnormal (irregular)	14	6.6	197	93.4	39	18.5	172	81.5	χ^2^ **=11.29; p=0.001** *
Menstrual bleeding duration										
	Abnormal (≤2 days or ≥8 days)	9	15.3	50	84.7	8	13.6	51	86.4	χ^2^=0.000; p=1
Perceived amount of bleeding										
	Abnormal (intensive/very intensive)	12	8.8	125	91.2	23	16.8	114	83.2	χ^2^=3.23; p=0.072
Heavy/very heavy menstrual bleeding days										
	Abnormal (3–10 days)	12	10.4	103	89.6	17	14.8	98	85.2	χ^2^=0.000; p=0.424
Perceived menstrual pain of bleeding										
	Abnormal (severe/very severe)	12	5.7	197	94.3	38	18.2	171	81.8	χ^2^ **=12.50; p**≤**0.001** *
Changing pads (less than 2 h)										
	Abnormal (usually and always)	9	7.1	118	92.9	21	16.9	106	83.5	χ^2^ **=4.03; p=0.045** *
Clot passage in the menstrual cycle										
	Abnormal (clot passage positive)	17	7.0	226	93.0	39	16.0	204	84.0	χ^2^ **=8.82; p=0.003** *
Using double pads										
	Abnormal (double pad use positive)	10	8.3	111	91.7	29	24.0	92	76.0	χ^2^ **=8.76; p=0.003** *
Blood overflow from pads into the bed/clothes										
	Abnormal (blood overflow positive)	15	5.8	242	94.2	48	18.7	209	81.3	χ^2^ **=17.96; p**≤**0.001** *

The age variable was removed because there were not enough students (n=7). Row percentage was used.

* p<0.05. Statistically significant values are denoted in bold.

## DISCUSSION

Our research examined the effects of education on adolescents’ knowledge of normal menstruation characteristics and their behaviors of seeking treatment in cases of deviation from normal. Our research determined that 6 out of every 10 adolescents received information about menstruation before menstruation, but only 7.8% received this information from a health professional. Studies have shown that the rate of adolescents receiving information about menstruation varies between 2.8 and 76.1%^17,18^. It has been reported that adolescents do not have sufficient information about menstruation and are unprepared for this process^17^. Improving high school students’ knowledge about sexually transmitted diseases, human papillomavirus (HPV), and vaccination, as well as increasing their knowledge about menstrual characteristics, may contribute to improving the health of adolescents^19,20^. Although there are differences in the rates of receiving information according to the development level of countries in the studies, it has been determined that the level of knowledge of adolescents about menstruation is not at the desired level, as in our study. This situation shows that health professionals should provide education on menstruation in a way that will eliminate the knowledge gap among adolescents.

Although menstrual problems are common^5^ and if these disorders are not treated, various health problems develop^8^ and reduce the quality of life of adolescents^21,22^. It has been reported that treatment-seeking behaviors on the subject remain inadequate^23^. A study conducted in Turkey reported that 11.8% of the adolescents were able to notice abnormal conditions related to their menstruation and relatively fewer people in this group (6.5%) showed treatment-seeking behavior^24^. Similar to all the study results mentioned above, our research also found that before receiving education about menstruation the rate of applying to a physician to seek treatment due to complaints experienced before and after menstruation was low.

In our study, after receiving education about menstruation, the rate of adolescents consulting a physician due to menstrual complaints decreased from 25.7 to 13.4%. However, the rate of adolescents reporting heavy and very HMB consulting a physician increased from 4.9 to 17.6%. Since no study was found showing the change in the status of applying to a physician or seeking treatment after receiving education about menstruation, the results of our research findings could not be discussed. However, our results show that education increases adolescents’ awareness in identifying their own HMB but is insufficient in increasing their level of awareness about their menstrual complaints. Therefore, in order to increase the awareness of adolescents about menstrual complaints, it is thought that education on the subject should be repeated at regular intervals and studies should be conducted to qualitatively examine the reasons for not seeking treatment.

In our research, it was determined that after the education, the majority of the normal characteristics related to menstruation were correctly known at 70% and above. For this reason, it is recommended that education on menstruation be given repeatedly and that health professionals provide consultancy when necessary, in order to increase the rate of correct knowledge of the students after the education and to make the information more memorable.

Studies have shown that education about menstruation has a positive effect on adolescents’ treatment-seeking behaviors. Similar to the results of this study^10,25^, our study found that adolescents who thought that they had heavy and very HMB were significantly more likely to seek treatment from a physician. However, our research has determined that those who experience complaints before and after menstruation are less likely to seek treatment from a physician after receiving the education compared to before receiving the education. It was determined that after the education, adolescents’ behavior to seeking treatment from a physician increased in all cases of deviation from normal, except for abnormality in the duration of menstrual bleeding. This situation shows that the education provided increased the awareness of adolescents about deviations from normal regarding menstruation.

### Strengths and limitations of the study

Studies examining the effect of education on adolescents’ knowledge of normal menstrual characteristics and treatment-seeking behaviors in cases of deviation from normal are limited. Therefore, it is thought that the information obtained from our study will contribute to the literature. Our strength is that it is the first quasi-experimental study on this subject. However, this also constitutes our limitation since there are no other studies to compare our research results. Since the data are based on students’ self-reports, it may create bias. Also, since our study was limited to students studying in a high school, the results of the study can only be generalized to this population.

## CONCLUSION

In our study, it was determined that the education provided increased the level of knowledge of adolescents about menstrual characteristics and their awareness of deviations from normal and had a positive effect on the treatment-seeking behavior of those with HMB. It is thought that it would be beneficial to repeat the education at regular intervals to increase the awareness of adolescents about menstrual irregularities.
